# A quantitative technique to create a femoral tunnel at the averaged center of the anteromedial bundle attachment in anatomic double-bundle anterior cruciate ligament reconstruction

**DOI:** 10.1186/1471-2474-14-189

**Published:** 2013-06-15

**Authors:** Shuken Kai, Eiji Kondo, Nobuto Kitamura, Yasuyuki Kawaguchi, Masayuki Inoue, Andrew A Amis, Kazunori Yasuda

**Affiliations:** 1 The Department of Sports Medicine and Joint Surgery, Hokkaido University Graduate School of Medicine, Sapporo, Hokkaido, Japan; 2 The Department of Orthopaedic Surgery, NTT East Japan Sapporo, Sapporo, Hokkaido, Japan; 3 The Department of Mechanical Engineering, Imperial College London, London, England, United Kingdom

**Keywords:** Anterior cruciate ligament, Anatomic reconstruction, Anteromedial bundle, Femoral tunnel, Footprint attachment location

## Abstract

**Background:**

In the anatomic double-bundle ACL reconstruction, 2 femoral tunnel positions are particularly critical to obtain better clinical results. Recently, a few studies have reported quantitative identification methods for posterolateral (PL) bundle reconstruction. Concerning anteromedial (AM) bundle reconstruction, however, no quantitative clinically available methods to insert a guide wire at the center of the direct attachment of the AM mid-substance fibers have been reported to date.

**Methods:**

First, we determined the center of the femoral attachment of the AM mid-substance fibers using 38 fresh frozen cadaveric knees. Based on this anatomical sub-study, we developed a quantitative clinical technique to insert a guide wire at the averaged center for anatomic double-bundle ACL reconstruction. In the second clinical sub-study with 63 patients who underwent anatomic ACL reconstruction with this quantitative technique, we determined the center of an actually created AM tunnel. Then, we compared the results of the second sub-study with those of the first sub-study to validate the accuracy of the quantitative technique. In both the sub-studies, we determined the center of the anatomical attachment and the tunnel outlet using the “3-dimensional clock” system. The tunnel outlet was evaluated using the “transparent” 3-dimensional computed tomography.

**Results:**

The averaged center of the direct attachment of the AM bundle midsubstance fibers was located on the cylindrical surface of the femoral intercondylar notch at “10:37” (or “1:23”) o’clock orientation in the distal view and at 5.0-mm from the proximal outlet of the intercondylar notch (POIN) in the lateral view. The AM tunnel actually created in ACL reconstruction was located at “10:41” (or “1:19”) o’clock orientation in the average and at 5.0-mm from the POIN. There was no significant difference between the 2 center locations.

**Conclusions:**

The quantitative technique enabled us to easily create the femoral AM tunnel at the averaged center of the direct attachment of the AM bundle midsubstance fibers with high accuracy. This study reported information on the geometric location of the femoral attachment of the AM bundle and a clinically useful technique for its anatomical reconstruction.

## Background

The anterior cruciate ligament (ACL) consists of two main bundles, the anteromedial (AM) and posterolateral (PL) bundles, which have different functions [1-6]. Anatomical studies have shown that the attachment of the AM and PL bundles can be divided into two parts: the direct attachment of the mid-substance fibers, and a wide fan-like attachment of the extension fibers posterior to the mid-substance fibers [7-10]. Biomechanical studies have shown advantages of anatomic double-bundle ACL reconstruction, in which 2 femoral tunnels were created at the center of the direct attachment of the AM and PL mid-substance fibers, respectively [11-15]. In the clinical field, many clinical studies have reported that the anterior and/or rotatory stability of the knee was significantly better with the anatomic double-bundle ACL reconstruction than with the single-bundle reconstruction [16-22]. However, the utility of the anatomic double-bundle reconstruction has not yet been established [23-26].

In the anatomic double-bundle ACL reconstruction, 2 femoral tunnel positions are particularly critical to obtain better clinical results [22,27]. Therefore, it is important to develop a clinically available quantitative method to precisely insert a guidewire at the center of the direct attachment of the AM and PL mid-substance fibers. Recently, a few studies have reported quantitative identification methods for PL bundle reconstruction [15,28,29]. Concerning AM bundle reconstruction, however, no quantitative clinically available methods to insert a guidewire at the center of the direct attachment of the AM mid-substance fibers have been reported to date. Several anatomical studies have quantitatively measured the location of the center of the AM bundle attachment using 2-dimensional measurement systems, such as the quadrant grid system [29-32]. However, those systems cannot be clinically applicable to arthroscopic surgery. Recently, it has been pointed out that the Resident’s ridge is located at the anterior border of the ACL attachment [8,33]. However, no studies have shown a quantitative method to insert a guidewire using the ridge as a reference in anatomical AM bundle reconstruction. Thus, at this point in time, surgeons have to subjectively determine the insertion point of a guidewire in an arthroscopic visual field to anatomically reconstruct the AM bundle. This fact may result in the wide variation of the location of the femoral tunnel created for AM bundle reconstruction reported in the literature [8,15,28,34,35].

Thus, we intended to develop a clinically available quantitative method to insert a guidewire at the averaged center of the direct attachment of the AM mid-substance fibers. Previous anatomical studies have shown that the AM mid-substance fibers attach on the lateral femoral condyle close to the “proximal outlet of the intercondylar notch (POIN)”, which appears to be circular in the axial view [7,9,36]. We have defined this 3-dimensional coordinate system as “3-dimensional clock system”. There is a strong possibility that the 3-dimensional clock system is useful in ACL reconstruction surgery to quantitatively locate a femoral tunnel at the center of the AM bundle attachment. However, this possibility has not been verified in clinical studies.

Thus, we have conducted a multi-disciplinary study to develop a quantitative technique in order to create a femoral tunnel at the averaged center of the AM bundle attachment in anatomic double-bundle ACL reconstruction, using the 3-dimensional clock system. This study is composed of anatomic and clinical sub-studies. The purpose of the first anatomical sub-study using 38 cadaveric knees is to determine the averaged location of the center of the femoral attachment of the AM mid-substance fibers using the 3-dimensional clock system in observation from the distal direction. Then, based on the results of the first sub-study, we have developed a quantitative technique to insert a guidewire at the averaged center of the AM bundle attachment in anatomic double-bundle ACL reconstruction. The purpose of the second clinical sub-study using 63 patients is to validate the accuracy of this quantitative method. The first specific hypothesis of this study is that the averaged center of the direct attachment of the AM bundle midsubstance fibers may be located at a specific o’clock orientation and at a fixed distance from the POIN in the 3-dimensional clock system. The second hypothesis is that the averaged center of the femoral AM tunnel actually created in the patients using the developed technique may be identical to the averaged center of the above-described anatomical AM bundle attachment.

## Methods

### The first anatomic sub-study

#### Study design

In this study, anatomic terminology will be used to describe positions of the knee in extension: that is proximal–distal, anterior–posterior, and medial-lateral.

**Figure 1:**
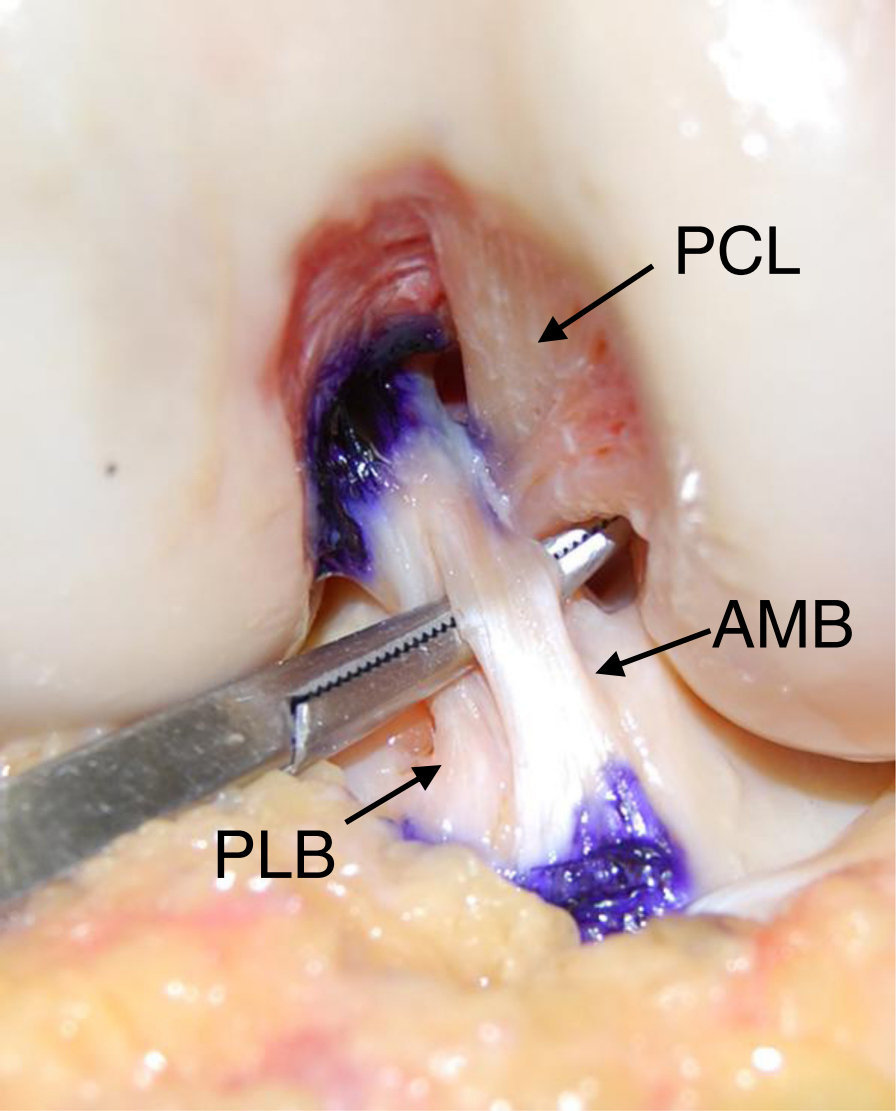
**The photograph of the AMB and PLB of the ACL in the axial view.** The ACL was bluntly separated into the two bundles. Note that the AM bundle attaches on the cylindrical surface of the femoral intercondylar notch around its proximal/posterior outlet. PCL: Posterior cruciat*e* ligament, AMB: Anteromedial bundle, PLB: Posterolateral bundle.

Thirty eight fresh frozen cadaveric knees (mean age, 61 years; range, 31–72 years) were obtained from the International Institute for the Advancement of Medicine (Jessup, Pennsylvania) with informed consent and permission from the institutional review board clearance in our institute. The knees were stored at −20 degrees Celsius were thawed a day before experimentation and kept moist with water spray. The femur and tibia were cut approximately 20 cm from the joint line. We removed all muscles and tendons around the knee joint as well as the joint capsule. We carefully removed the synovial membrane around the ACL. The shape and function of the AM and PL bundles were observed at various angles of knee flexion. The 2 bundles were identified on the basis of their orientations and tension under anterior drawer forces and internal and external torque. Then, the ACL was bluntly separated into the two bundles (Figure 1). The ligaments were transected and the femur was disarticulated from the tibia. Then, each bundle of the ACL was transected with a knife so that 1-mm long ligament fibers were left at the femoral attachment. We perpendicularly inserted a marker pin, which had a spherical red-colored head having a diameter of 2 mm, at the center of the femoral attachment of the AM mid-substance fibers.

**Figure 2:**
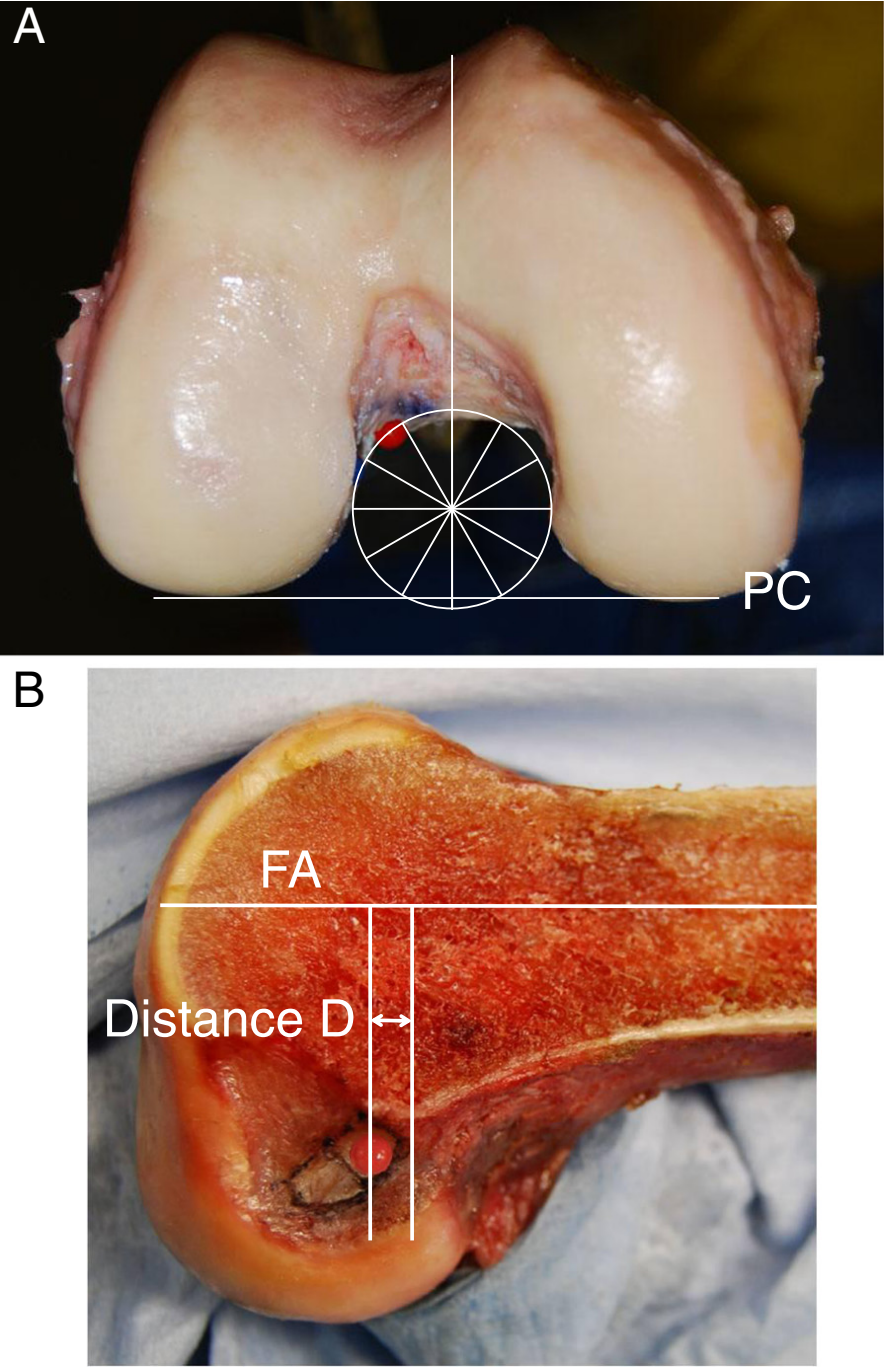
**The photographs of the femoral notch in the axial view and the lateral view. **On a photograph taken in the axial view (**A**), we drew a circle superimposed onto the cylindrical roof and lateral wall around the POIN. Then, we drew a diameter of this circle which was perpendicular to the posterior condyle (PC) line. Then, the center of the red marker was measured with the so-called “clock” system. On a photograph taken in the lateral view (**B**), we drew a reference line of the femoral shaft (FS) axis. Then, we drew 2 lines, which were perpendicular to the reference line and passing the center of the femoral attachment of the AM mid-substance fibers (a red marker) and the POIN, respectively. A distance between the 2 lines was defined as Distance D.

The femur was fixed to a custom-made stainless steel stand with a clamp so that the axis of the distal femoral shaft was horizontal. A single-lens reflex digital camera (Nikon D40, Tokyo, Japan) was secured to a stand located 1 m away from the femur. A photograph of the femoral notch was taken in an accurate axial-anterior view (Figure 2A). Then, the medial femoral condyle was removed with a bone saw to laterally observe the ACL attachment site, as described by Zavras and Amis [37]. The digital camera was secured to a stand located 1 m away from the femur. A photograph of the lateral femoral condyle was taken in an accurate lateral view (Figure 2B).

### Measurement

We measured the center of the femoral attachment of the AM mid-substance fibers using the 3-dimensional clock system, according to Edwards et al [36]. On the picture taken in the distal view, we superimposed a circle onto the cylindrical roof and lateral wall at the POIN. Then, we drew a diameter of this circle which was perpendicular to the posterior condyle line. The 6 o’clock and 12 o’clock orientation was defined by this diameter. Then, the center of the red marker was measured with the so-called “clock” expression system (Figure 2A). On the picture in the lateral view, we drew a reference line of the axis of the distal femoral shaft. Then, we drew 2 lines, which were perpendicular to the reference line and passing through the center of the red marker and the POIN, respectively. The distance between the 2 lines, which was defined as Distance D, was measured (Figure 2B). Thus, the center of the AM bundle attachment was 3-dimensionally determined by 2 measured values, the “time” and the distance D.

**Figure 3:**
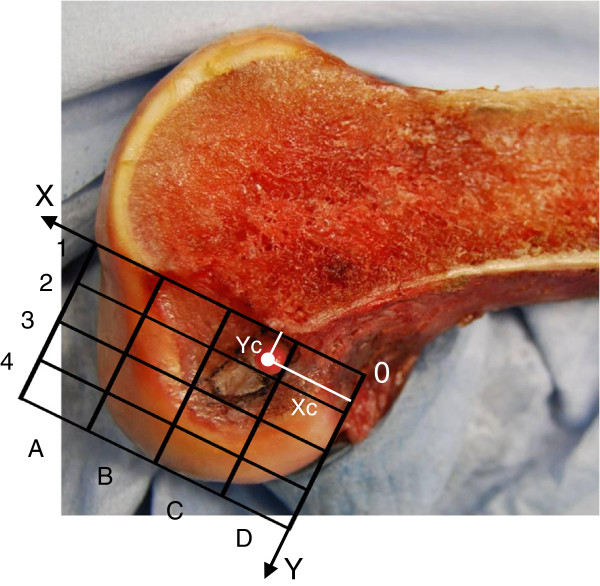
**Positions of the centers of the AM tunnel within the measurement grid on the lateral view photograph of the lateral femoral condyle.** We superimposed a measurement grid [30] onto the lateral view photograph, using the roof of the intercondylar notch as a reference line. Then, the grid was divided into 16 zones. We also defined the X-Y coordinate system on this grid. The cordinate of the center of the femoral attachment of the AM mid-substance fibers, (Xc, Yc), was defined as follows: Xc was defined as the distance between the center and the Y axis, and Yc was defined as the distance between the center and the X axis.

Additionally, to compare the results of the present study with those in the previously reported studies, we superimposed a measurement grid [30] onto the lateral view photograph, using the roof of the intercondylar notch as a reference line. Then, the grid was divided into 16 zones (Figure 3). We also defined the X-Y coordinate system on this grid. Namely, the roof line of the intercondylar notch was defined as an X-axis. The most proximal line of the grid lines which were perpendicular to the X-axis was defined as a Y-axis. Then, on this X-Y coordinate system, the coordinate of the center of the femoral attachment of the AM mid-substance fibers, (Xc, Yc), was defined as follows: Xc was defined as the distance between the center and the Y axis, and Yc was defined as the distance between the center and the X axis (Figure 3).

### The second clinical evaluation sub-study

#### The newly developed quantitative technique to insert a guide wire

**Figure 4:**
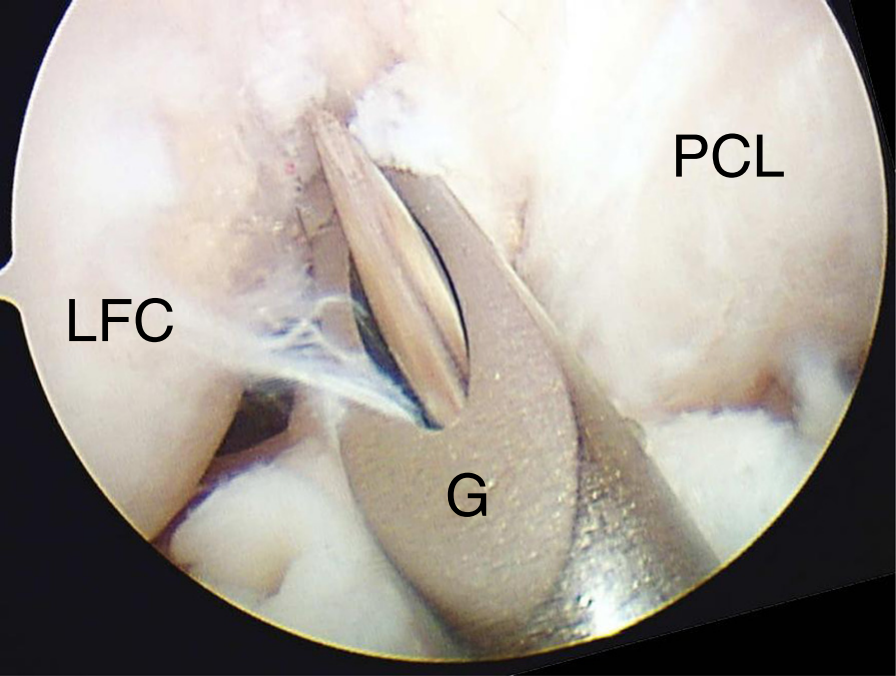
**An offset guide position in arthroscopic view by use of lateral infrapatellar portal.** A quantitative surgical technique to insert a guide wire for AM bundle reconstruction. Through the tibial tunnel, a hook-shaped tip of a 5-mm offset guide (G) was set at the “over-the-top” portion of the lateral condyle at 90 – 100 degrees of knee flexion, and then, slightly rotated so that the tip of the guide wire was aimed at the “1:30” or “10:30” orientation. PCL: Posterior cruciate ligament, LFC: Lateral femoral condyle.

Based on the results of the first anatomical sub-study, we developed a clinically available quantitative technique using the 3-dimensional clock system to insert a guide wire at the averaged center of the femoral attachment of the AM mid-substance fibers. The results of the first sub-study showed that the averaged center was located at “1:23” or “10:37” o’clock orientation. In the actual technique, however, we aimed at “1:30” or “10:30” orientation, because this degree, an eighth of a circle, could be easily detected by a surgeon in the arthroscopic visual field and the difference was clinically negligible. Thus, the concept of this quantitative technique was to insert the guide wire at the point with “1:30” or “10:30” o’clock orientation and a distance D of 5 mm from the POIN on the 3-dimensional clock system. To achieve this concept, we used the following technique: We commonly used the transtibial procedure, the essence of which is to drill a tibial AM tunnel prior to femoral tunnel creation so that the tibial tunnel axis approximately passed at the center of the femoral attachment of the AM mid-substance fibers [15,18,22,35]. Therefore, through the tibial tunnel, we introduced a 5-mm offset guide (Transtibial Femoral ACL Drill Guide, Arthrex, Inc., Naples, FL, USA) into the joint cavity, and easily set the hook-shaped tip of this guide at the “over-the-top” portion of the lateral condyle at 90 – 100 degrees of knee flexion. Keeping the hook at this point, we rotated the offset guide so that the tip of a guide wire inserted through the guide was aimed at the “1:30” or “10:30” o’clock orientation in the arthroscopic visual field (Figure 4). It is critical to manually force the guide to be sufficiently rotated, although this is not the usual method for use of this guide. Then, we inserted a guide wire into the femur.

### Patients and study design

We conducted a prospective evaluation study with patients who underwent anatomic double-bundle ACL reconstruction using the above-described quantitative technique to insert a guide wire for AM bundle reconstruction. In subjects who accepted to participate in this study, we took the “transparent 3-dimensional computed tomography” (T-3D CT) of the knee in the extended position based on a previous study [38] one to two weeks after surgery. The T-3D CT images were taken using a Light Speed Ultra (8Das) Helical scan, GE, Milwaukee, USA, and the images were processed by using a workstation (ZIO M900 Quadra, Tokyo, Japan). By condensing the CT value (minimum threshold 100–160), a number of pixels on the bone surface remained, but the opacity of all the other bone tunnel pixels decreased to produce highly transparent (volume-rendering) processed T-3D CT images. The dose of radiation to the knee joint was approximately 6 mGy, which was the same as that in standard CT imaging. During scanning, the patients wore protective clothing and only the knee was exposed. All patients were informed that, if they did not want to participate in this study, we did not take T-3D CT images. This clinical study design had been accepted by the institutional review board in our hospital prior to commencement, based on the above-described study design.

Thus, 63 patients (43 men and 20 women) took part in this prospective study in 2009. Their mean age was 28 years (ranging from 13 to 55 years). The mean interval between time of the injury and the operation was 2.4 months (ranging from 1 to 36 months). Meniscus injuries were found in 35 knees. Of these, 10 menisci were partially resected, and the remaining 25 were sutured. One senior orthopedic surgeon (##) performed all operations. Anatomic double-bundle ACL reconstruction was carried out with the previously reported procedure [15,18] including the above-described quantitative surgical technique to insert a guide wire.

### ACL reconstruction procedure

We performed anatomic double-bundle ACL reconstruction using the transtibial technique, in which two tibial tunnels were created so that each tunnel axis line passed through the center of the femoral attachment of the midsubstance fibers of the AM or PL bundle [15,18]. To create such tunnels, we used a Wire-navigator (Smith & Nephew Endoscopy, Japan). The “Navi-tip” portion of this devise was introduced into the joint cavity through the medial infrapatellar portal. The surgeon held the tibia at 90 degrees of knee flexion, keeping the femur horizontal. The tibial indicator of the Navi-tip was placed at the center of the PL or AM bundle attachment on the tibia. Then, keeping the tibial indicator at this point, we aimed the femoral indicator at the center of the femoral attachment of the midsubstance fibers of the AM or PL bundle. Specifically for the AM bundle reconstruction, we then aimed the femoral indicator at the center of the femoral attachment of the AM bundle, which was determined in the first sub-study, at 90 degrees of knee flexion. Then, the direction of the Wire Sleeve and the insertion point of a guide wire on the AM aspect of the tibia were automatically decided. After a guide wire was drilled into the tibia, two tibial tunnels were created with a cannulated drill corresponding to the measured diameter of the prepared tendon graft.

Using the above-described quantitative technique, we inserted a wire through the tibial tunnel into the averaged center of the femoral attachment of the mid-substance fibers of the AM bundle. Using this wire as a guide, a tunnel was made with a 4.5-mm cannulated drill. The length of the tunnel was measured with a scaled probe. Then, the portal for the arthroscope was changed to the medial infrapatellar portal. The surgeon again held the tibia at 90 degrees of knee flexion, keeping the femur horizontal. The surgeon inserted a guide wire at the center of the PL bundle attachment on the femur through the tibial tunnel. A 4.5 mm-diameter tunnel was drilled, and its length was measured in the same manner. Finally, 2 sockets were created for the AM and PL bundles, respectively, with cannulated drills for the EndoButton fixation system (Smith & Nephew Endoscopy), the diameters of which were matched to the 2 grafts. The doubled hamstrings tendon grafts were fixed with an Endobutton-CL-BTB at the femoral end and with a tape and two staples at the tibial end.

### Measurement of the CT image

**Figure 5:**
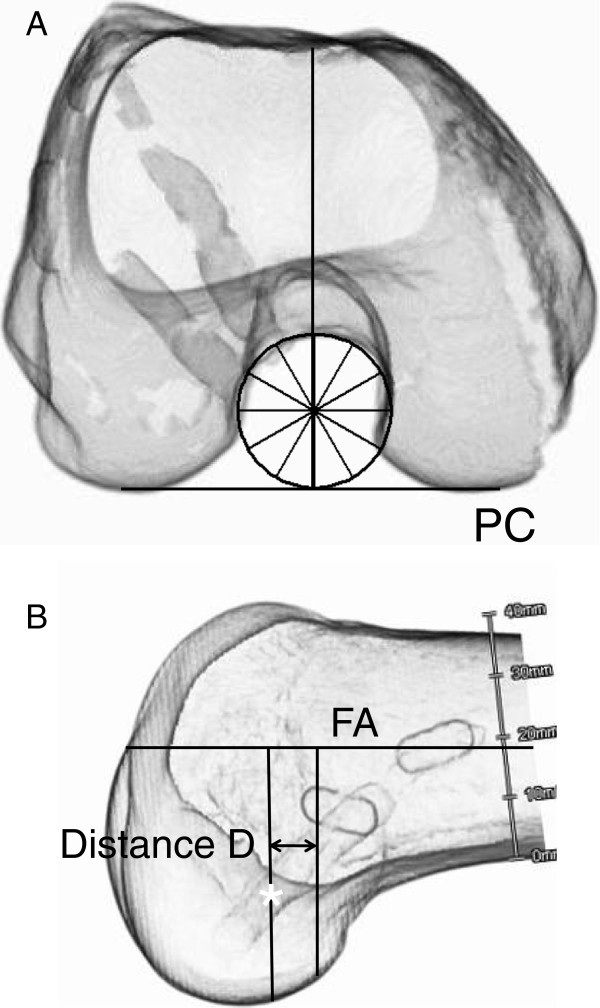
**The T-3D CT images of the femoral notch in the axial view and the lateral view.** On a photograph taken in the axial view (**A**) and the lateral view (**B**), the center of an actually created AM tunnel was measured by using the same 3-dimensional clock system as used in the first sub-study (PC and FA: See Figure 4).

**Figure 6:**
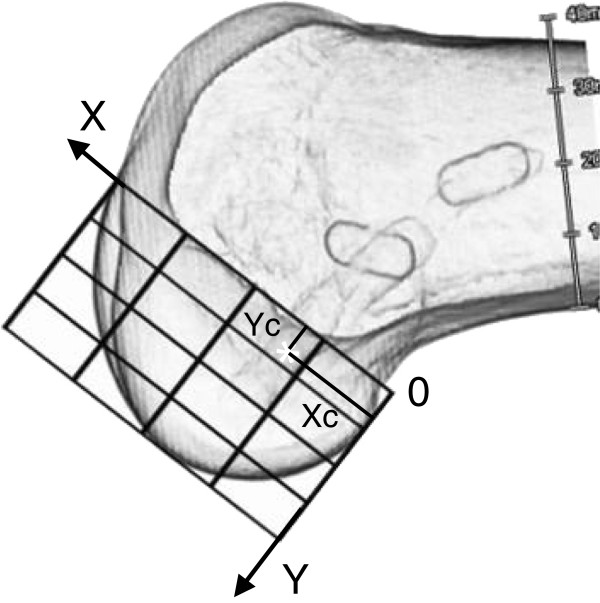
**Positions of the centers of the AM tunnel within the measurement grid on the T-3D CT image of the lateral femoral condyle.** The same measurement grid as used in the first sub-study was superimposed onto the T-3D CT image taken in the lateral view. On this grid, the coordinate of the center of the femoral AM tunnel, (Xc, Yc), was defined in the same manner as used in the first sub-study (See Figure 5).

The greatest feature of the T-3D CT image was that we could obtain an accurate axial view of the intercondylar notch (Figure 5A). The lateral view was obtained by overlapping the medial and lateral femoral condyles in the image, according to Cole et al [39] (Figure 5B). The T-3D CT images simultaneously depicted the femoral tunnels and the outer margin of the femoral cortex. On the axial and lateral view images, the center of the actually created AM tunnel was measured by using the above-described 3-dimensional clock system (Figure 5A and B), as in the first sub-study. In addition, to compare the results between the first and second sub-studies, we superimposed the same measurement grid as used in the first sub-study onto the lateral T-3D CT image, using the roof of the intercondylar notch as a reference line (Figure 6). On this grid, we defined the coordinates of the center of the femoral AM tunnel, (Xc, Yc), as in the first sub-study.

### Statistical analysis

Statistical analysis used the Student’s t test and the chi-square test to compare the averaged center of the actually created AM tunnels, which was evaluated in the second sub-study with the averaged center of the normal AM bundle attachment on the femur, which was determined in the first sub-study. In this study, the power values were 0.96 or more.

## Results

### The first anatomical sub-study

In the axial view from the distal direction, the averaged center of the direct attachment of the AM bundle midsubstance fibers was located at “10:37” (or “1:23”) o’clock orientation with the standard deviation of 20 minutes (Table 1). In the lateral view, the averaged Distance D was 5.0 mm with the standard deviation of 1.2 mm (Table 1). According to the above-defined grid system, the center of the direct attachment of the AM bundle midsubstance fibers was located in zone C1 for 18 knees (47.4%), in zone D1 for 14 knees (36.8%), in zone C2 of 5 knees (13.2%), and in zone D2 for 1 knee (2.6%). On this grid, the mean Xc coordinate of the averaged center of the AM bundle attachment was 25.8 (%), and the mean Yc coordinate of that was 20.7 (%).

**Table 1:** Summary of the 2 sub-studies: the anatomical study of the averaged center of the AM bundle attachment on the femur (Sub-study I) and the averaged center of the actually created femoral tunnel for AM bundle reconstruction (Sub-study II). Each value shows “the average (Standard deviation)”

	**Sub-study I (n=38)**	**Sub-study II (n=63)**	***P*–value**	**Power**
Orientation (o’clock)	10:37 (0:20)	10:41 (0:18)		p=0.2998	1.0
Distance D (mm)	5.0 (1.2)	5.0 (1.1)	p=0.9496	1.0	
Xc (%)	25.8 (3.1)	26.7 (1.9)	p=0.0841	0.97	
Yc (%)	20.7 (5.2)	19.6 (4.9)	p=0.2722	0.96	

The o’clock orientation shows the value in the right knee.

### The second clinical sub-study

In the axial distal view, the averaged center of the femoral AM tunnel was located at “10:41” (or “1:19”) o’clock orientation (Table 1). In the lateral view, the averaged Distance D was 5.0 mm (Table 1). In comparison of the results between the first and second studies, there were no significant differences in the o’clock orientation and D values (*P*=0.2998, and *P*=0.9496, respectively) between the centers in the 2 sub-studies. According to the above-defined grid system, the center of the direct attachment of the AM bundle midsubstance fibers was located in zone C1 for 45 knees (71.4%), in zone D1 for 11 knees (17.5%), in zone C2 for 5 knees (7.9%), and in zone D2 for 2 knees (3.2%). There was no significant difference between the two sub-studies (Chi-square test, *P*=0.0907). On this grid, the mean Xc coordinate of the averaged center of the AM bundle attachment was 26.7 (%), and the mean Yc coordinate of that was 19.6 (%) (Table 1). In comparison of the results between the first and second studies, there were no significant differences in the Xc and Yc values (*P*=0.0841 and *P*=0.2722, respectively; Table 1).

## Discussion

The most important finding of the present study was that this first anatomical sub-study clarified where the averaged center of the direct attachment of the AM bundle midsubstance fibers was located on the cylindrical surface of the femoral intercondylar notch around its proximal outlet. Namely, the averaged center was located at “10:37” (or “1:23”) o’clock orientation in the distal view and at 5.0-mm from the POIN in the lateral view. Edwards et al [36] reported that the averaged center was located at 1:30 or 10:30 o’clock orientation and 4.7 mm to the POIN on the same 3-dimensional clock system. The results of these 2 studies observed from distal and proximal directions, respectively, are similar. Therefore, the present study results are reliable, and provide more direct information to arthroscopic AM bundle reconstruction, which was performed in the distal view. Namely, the results enabled us to develop the quantitative guideline to insert a guide wire at the averaged center of the direct attachment of the AM bundle midsubstance fibers for anatomic double-bundle ACL reconstruction.

On the other hand, previous anatomical studies using the 2-dimensional coordinate systems have reported different locations of the averaged center of the direct attachment of the AM bundle midsubstance fibers: [28,29,31,32] The mean Xc coordinate varied from17.8% to 31.9%, and the mean Yc coordinate varied from 22.3% to 26.9%. In the present study, the averaged center, which was located 3-dimensionally, was represented in the 2-dimensional coordinate system as follows: The mean Xc coordinate was 25.8%, and the mean Yc coordinate was 20.7%. The variations of the Xc and Yc values between the studies are considered to be due to the differences in definition of the X-Y coordinate system as well as the definition of the lateral view of the knee. In addition, we should note that the 2-dimensinal coordinate systems cannot be used when a surgeon inserts a guide wire into the averaged center of the AM bundle attachment in an arthroscopic visual field. In contrast, the 3-dimensional clock system used in the present study is clinically useful because it can be used easily by surgeons during arthroscopic ACL reconstruction.

In our clinical sub-study, there was no significant difference between the averaged center of the femoral AM tunnel actually created in the 63 patients and the averaged center of the anatomical AM bundle attachment. Because the standard deviation of the center of the created tunnel was quite little (18 minutes around the ‘clock’ and 1.1 mm from the POIN), we believe that the accuracy of this technique was sufficiently high, and that the theoretical concept of this technique was realized in anatomic double-bundle ACL reconstruction. This sub-study demonstrated that the quantitative technique is useful for anatomical AM bundle reconstruction. The accuracy of this technique is considered to be supported by the following 2 factors. First, the 3-dimensional clock system used in this study is suitable to express the center of the 3-dimensional attachment of the AM bundle, which is on the cylindrical surface of the femoral intercondylar notch [28,29,31,32]. Secondly, our surgical technique is quantitative, using an offset guide which matched the measured Distance D. It was quasi-quantitative to estimate the “1:30” o’clock orientation in an arthroscopic visual field, because it was easy for experienced surgeons to estimate an eighth of a circle. We believe that this quantitative technique is simple and clinically useful to precisely create the femoral AM tunnel at the averaged center of the direct attachment of the AM bundle midsubstance fibers. The concept of this technique using the 3-dimensional clock system can be applicable to all procedures in which surgeons intend to create a femoral AM tunnel at the averaged center of the AM bundle attachment. In addition, this surgical concept may be applicable to a navigation or robotic system for anatomic ACL reconstruction in the near future.

We have used the transtibial technique in our anatomic double-bundle ACL reconstruction procedure. We should note that the essence of the transtibial procedure is to drill a tibial tunnel so that the tunnel axis was aligned approximately towards the targeted point on the femur [35]. Once the tibial AM tunnel was appropriately created, the above-described quantitative technique to create the femoral AM tunnel was easy. However, if the AM tibial tunnel was inappropriately created, the present technique may be difficult. Therefore, it is important to create the appropriate tibial AM tunnel. To create such a tibial tunnel, some devices are reported to be helpful for surgeons [11,15,35]. To achieve this concept with high reproducibility, we developed the guidewire navigator [11,15,35]. This hole-in-one guide allowed surgeons to imagine the intra-articular position and the direction of each bundle to be reconstructed, and to determine the extraarticular insertion point on the tibia for a guidewire, which was located directly on the axis of the bundle. In addition, the authors believe that our previously reported data [40] on the axis of the tibial tunnels is of value for surgeons who perform anatomic double-bundle ACL reconstruction, because these angle values may provide references for the creation of appropriate tunnels in the tibia for surgeons who want to follow this procedure. It is also a critical technique to force the offset guide to be sufficiently rotated so that the guidewire aims at the 1:30 or 10:30 orientation. Although we could successfully rotate the currently used offset guide, improvement of the guide may be necessary in the near future for every surgeon to more easily insert a guidewire. Then, the 3-dimensional data concerning the center of the AM bundle attachment, obtained from the present study, will contribute to the improvement. If a surgeon use an AM portal in this double-bundle procedure, this 3-dimentional data will be helpful to create the femoral AM tunnel. However, a surgeon should take an attention for the difference of knee angle between the transtibial and AM portal techniques, because the 3-dimensional clock circle was located on the femoral intercondylar notch which was perpendicular to the axis of the femoral shaft in this study. On the other hand, this technique cannot be applied so easily to the PL tunnel creation. Because the PL bundle attaches on the relatively flat postero-distal surface of the femoral intercondylar notch, the center of the PL bundle attachment can more easily be defined using a 2-dimensional coordinate system [38].

In previous literature, a few studies reported the position of the femoral AM tunnel actually created in patients, using 2-dimensional systems [12,31,33]. The results vary widely. The reasons may include that the previous studies did not use any quantitative techniques to insert a guide wire to create the femoral AM tunnel. In the existing literature, no studies have evaluated the femoral AM tunnel position with the 3-dimensional system. Therefore, we cannot compare the results of the present study with those of the previous literature.

In our surgical strategy, we intended to create the femoral AM tunnel at the averaged center of the direct attachment of the AM bundle midsubstance fibers in every patient. There may be some criticisms as to our strategy. The first criticism is that we should not create the femoral AM tunnel at the averaged center, but create it at the real center of an individual patient. This criticism is theoretically correct. However, it is impossible to precisely identify the real center of the AM bundle attachment in an individual patient. In contrast, the standard deviation of the averaged center of the AM bundle attachment was only 1.2 mm, which was sufficiently minimal in comparison with the relatively wide area of the AM bundle attachment. We believe that our quantitative strategy is clinically useful to precisely create the femoral AM tunnel at an acceptable point in every patient. The second criticism is that we should create the femoral AM tunnel between the resident’s ridge and the articular cartilage margin on the lateral femoral condyle [8,27]. This area includes the attachment of both mid-substance fibers and fan-like extension fibers. However, many biomechanical studies have shown that the femoral AM tunnel created at the center of the direct attachment of the AM bundle midsubstance fibers can restore the functions close to the normal AM bundle [11-13,41-45]. Therefore, we believe that it is reasonable to create the AM tunnel at the averaged center of the direct attachment of the AM bundle midsubstance fibers. The third criticism is that the distance D may be greater than 5 mm in patients who have a much larger knee (which is correlated to the body size) than our patients, who have a relatively narrow range between 150 cm and 179 cm of body height. However, even for a patient 185 cm tall, the 5 mm offset will only increase proportionally to 5.6 mm in relation to our set of knees based on donors around 165 cm tall.

## Conclusions

The anatomical study showed that the averaged center of the direct attachment of the AM bundle midsubstance fibers was located on the cylindrical surface of the femoral intercondylar notch around its proximal outlet at “10:37” (or “1:23”) o’clock orientation in the distal view and at 5.0-mm from the POIN in the lateral view.

Based on these data, we developed a technique to insert a guide wire at this point, using a 5-mm offset guide at the “over-the-top” portion of the lateral condyle, and we rotated the offset guide so that the tip of a guide wire inserted through the guide was aimed at the “1:30” or “10:30” o’clock orientation in the arthroscopic visual field.

The clinical study validated this method: the AM tunnel actually created in ACL reconstruction was located at “10:41” (or “1:19”) o’clock orientation in the average and 5.0-mm from the POIN. There was no significant difference between the averaged center of the femoral AM tunnel actually created in the 63 patients and the averaged center of the anatomical AM bundle attachment. The present study demonstrated that the quantitative technique is useful with the high accuracy for anatomical AM bundle reconstruction.

## Competing interests

We have no financial or non- financial competing interests. We do not hold or are not currently applying for any patents relating to the content of the manuscript.

## Authors’ contributions

SK performed all experiments. EK, KY, and AA designed the study, participated in the study, and drafted the manuscript. NK, MI and YK participated in designing the study and instructed CT measurements. All authors read and approved the final manuscript.

## Pre-publication history

The pre-publication history for this paper can be accessed here:

http://www.biomedcentral.com/1471-2474/14/189/prepub

## References

[B1] Amis AA Dawkins GP Functional anatomy of the anterior cruciate ligament Fiber bundle actions related to ligament replacement and injuries 1991 73 260 267 10.1302/0301-620X.73B2.2005151 2005151

[B2] Bach JM Hull ML Patterson HA Direct measurement of strain in the posterolateral bundle of the anterior cruciate ligament J Biomech 1997 30 281 283 10.1016/S0021-9290(96)00132-7 9119829

[B3] Gabriel MT Wong EK Woo SL Yagi M Debski RE Distribution of in situ forces in the anterior cruciate ligament in response to rotator loads J Orthop Res 2004 22 85 89 10.1016/S0736-0266(03)00133-5 14656664

[B4] Kurosawa H Yamakoshi K Yasuda K Sasaki T Simultaneous measurement of changes in length of the cruciate ligaments during knee motion Clin Orthop Relat Res 1991 265 233 240 2009664

[B5] Sakane M Fox RJ Woo SL Livesay GA Li G Fu FH In situ forces in the anterior cruciate ligament and its bundles in response to anterior tibial loads J Orthop Res 1997 15 285 293 10.1002/jor.1100150219 9167633

[B6] Yasuda K van Eck CF Hoshino Y Fu FH Tashman S Anatomic single- and double-bundle anterior cruciate ligament reconstruction, part 1: basic science Am J Sports Med 2011 39 1789 1799 10.1177/0363546511402659 21596902

[B7] Hara K Mochizuki T Sekiya I Yamaguchi K Akita K Muneta T Anatomy of normal human anterior cruciate ligament attachments evaluated by divided small bundles Am J Sports Med 2009 37 2386 2391 10.1177/0363546509340404 19940312

[B8] Iwahashi T Shino K Nakata K Otsubo H Suzuki T Amano H Nakamura N Direct anterior cruciate ligament insertion to the femur assessed by histology and 3-dimensional volume-rendered computed tomography Arthroscopy 2010 26 S13 20 10.1016/j.arthro.2010.01.023 20667684

[B9] Mochizuki T Muneta T Nagase T Shirasawa S Akita K Sekiya I Cadaveric knee observation study for describing anatomic femoral placement for two-bundle anterior cruciate ligament reconstruction Arthroscopy 2006 22 356 361 10.1016/j.arthro.2005.09.020 16581446

[B10] Siebold R Ellert T Metz S Metz J Femoral insertions of the anteromedial and posterolateral bundles of the anterior cruciate ligament: morphometry and arthroscopic orientation models for double-bundle bone tunnel placement-A cadaver study Arthroscopy 2008 24 585 592 10.1016/j.arthro.2007.12.008 18442692

[B11] Kondo E Merican AM Yasuda K Amis AA Biomechanical comparisons of knee stability after anterior cruciate ligament reconstruction between 2 clinically available transtibial procedures: anatomic double bundle versus single bundle Am J Sports Med 2010 38 1349 1358 10.1177/0363546510361234 20423987

[B12] Yagi M Wong EK Kanamori A Debski RE Fu FH Woo SL Biomechanical analysis of an anatomic anterior cruciate ligament reconstruction Am J Sports Med 2002 30 660 666 12238998 10.1177/03635465020300050501

[B13] Yamamoto Y Hsu WH Woo SL Van Scyoc AH Takakura Y Debski RE Knee stability and graft function after anterior cruciate ligament reconstruction A comparison of a lateral and an anatomical femoral tunnel placement 2004 32 1825 1832 10.1177/0363546504263947 15572308

[B14] Yasuda K Ichiyama H Kondo E Miyatake S Inoue M Tanabe Y An in vivo biomechanical study on the tension-versus-knee flexion angle curves of two grafts in anatomic double-bundle anterior cruciate ligament reconstruction: effects of initial tension and internal tibial rotation Arthroscopy 2008 24 276 284 10.1016/j.arthro.2007.08.031 18308178

[B15] Yasuda K Kondo E Ichiyama H Anatomic reconstruction of the anteromedial and posterolateral bundles of the anterior cruciate ligament using hamstring tendon grafts Anatomic and clinical studies. Arthroscopy 2004 20 1015 1025 10.1016/j.arthro.2004.08.010 15592229

[B16] Aglietti P Giron F Cuomo P Losco M Mondanelli N Single-and double-incision double-bundle ACL reconstruction Clin Orthop Relat Res 2007 454 108 113 17202919 10.1097/BLO.0b013e31802baaf4

[B17] Jarvela T Double-bundle versus single-bundle anterior cruciate ligament reconstruction: a prospective, randomize clinical study Knee Surg Sports Traumatol Arthrosc 2007 15 500 507 10.1007/s00167-006-0254-z 17216271

[B18] Kondo E Yasuda K Azuma H Tanabe Y Yagi T Prospective clinical comparisons of anatomic double-bundle versus single-bundle anterior cruciate ligament reconstruction procedures in 328 consecutive patients Am J Sports Med 2008 36 1675 1687 10.1177/0363546508317123 18490472

[B19] Muneta T Koga H Mochizuki T A prospective randomized study of 4-strand semitendinosus tendon anterior cruciate ligament reconstruction comparing single-bundle and double-bundle techniques Arthroscopy 2007 23 618 628 10.1016/j.arthro.2007.04.010 17560476

[B20] Siebold R Dehler C Ellert T Prospective randomized comparison of double-bundle versus single-bundle anterior cruciate ligament reconstruction Arthroscopy 2008 24 137 145 10.1016/j.arthro.2007.11.013 18237696

[B21] Yagi M Kuroda R Nagamune K Yoshiya S Kurosaka M Double bundle ACL reconstruction can improve rotational stability Clin Orthop Relat Res 2007 454 100 7 17091015 10.1097/BLO.0b013e31802ba45c

[B22] Yasuda K Kondo E Ichiyama H Tanabe Y Tohyama H Clinical evaluation of anatomic double-bundle anterior cruciate ligament reconstruction procedure using hamstring tendon grafts: Comparisons among 3 different procedures Arthroscopy 2006 22 240 250 10.1016/j.arthro.2005.12.017 16517306

[B23] Lewis PB Parameswaran AD Rue JP Bach BR Jr Systematic review of single-bundle anterior cruciate ligament reconstruction outcomes: a baseline assessment for consideration of double-bundle techniques Am J Sports Med 2008 36 2028 2036 10.1177/0363546508322892 18757764

[B24] Meredick RB Vance KJ Appleby D Lubowitz JH Outcome of single-bundle versus double-bundle reconstruction of the anterior cruciate ligament: a meta-analysis Am J Sports Med 2008 36 1414 1421 10.1177/0363546508317964 18508977

[B25] Streich NA Friedrich K Gotterbarm T Schmitt H Reconstruction of the ACL with a semitendinosus graft: a prospective randomized single-blinded comparison of double-bundle versus single-bundle technique in male athletes Knee Surg Sports Traumatol Arthrosc 2008 16 232 238 10.1007/s00167-007-0480-z 18193194

[B26] Yasuda K Tanabe Y Kondo E Kitamura N Tohyama H Anatomic double-bundle anterior cruciate ligament reconstruction Arthroscopy 2010 26 S21 34 10.1016/j.arthro.2010.03.014 20810091

[B27] van Eck CF Lesniak BP Schreiber VM Fu FH Anatomic single- and double-bundle anterior cruciate ligament reconstruction flowchart Arthroscopy 2010 26 258 268 10.1016/j.arthro.2009.07.027 20141990

[B28] Colombet P Robinson J Christel P Morphology of anterior cruciate ligament attachments for anatomic reconstruction: A cadaveric dissection and radiographic study Arthroscopy 2006 22 984 992 10.1016/j.arthro.2006.04.102 16952729

[B29] Zantop T Wellmann M Fu FH Petersen W Tunnel positioning of anteromedial and posterolateral bundles in anatomic anterior cruciate ligament reconstruction. Anatomic and radiographic findings Am J Sports Med 2008 36 65 72 17932407 10.1177/0363546507308361

[B30] Bernard M Hertel P Hornung H Cierpinski T Femoral insertion of the ACL Radiographic quadrant method 1997 10 14 21 9051173

[B31] Takahashi M Doi M Abe M Suzuki D Nagano A Anatomical study of the femoral and tibial insertions of the anteromedial and posterolateral bundles of human anterior cruciate ligament Am J Sports Med 2006 34 787 792 16452272 10.1177/0363546505282625

[B32] Tsukada H Ishibashi Y Tsuda E Fukuda A Toh S Anatomical analysis of the anterior cruciate ligament femoral and tibial footprints J Orthop Sci 2008 13 122 129 10.1007/s00776-007-1203-5 18392916

[B33] Ferretti M Ekdahl M Shen W Fu FH Osseous landmarks of the femoral attachment of the anterior cruciate ligament: an anatomic study Arthroscopy 2007 23 1218 25 10.1016/j.arthro.2007.09.008 17986410

[B34] van Eck CF Schreiber VM Mejia HA Samuelsson K van Dijk CN Karlsson J Fu FH “Anatomic” anterior cruciate ligament reconstruction: a systematic review of surgical techniques and reporting of surgical data Arthroscopy 2010 26 S2 12 10.1016/j.arthro.2010.03.005 20810090

[B35] Yasuda K Kondo E Kitamura N Kawaguchi Y Kai S Tanabe Y A pilot study of anatomic double-bundle anterior cruciate ligament reconstruction with ligament remnant tissue preservation Arthroscopy 2012 28 343 53 10.1016/j.arthro.2011.08.305 22154365

[B36] Edwards A Bull AMJ Amis AA The attachments of the anteromedial and posterolateral fibre bundles of the anterior cruciate ligament part 2: femoral attachment Knee Surg Sports Traumatol Arthrosc 2008 16 29 36 10.1007/s00167-007-0410-0 17957351

[B37] Zavras TD Amis AA Method for visualising and measuring the position of the femoral attachment of the ACL and ACL grafts in experimental work J Biomech 1998 31 387 390 10.1016/S0021-9290(98)00019-0 9672094

[B38] Inoue M Tokuyasu S Kuwahara S Tunnel location in transparent 3-dimensional CT in anatomic double-bundle anterior cruciate ligament reconstruction with the trans-tibial tunnel technique Knee Surg Sports Traumatol Arthrosc 2010 18 1176 83 10.1007/s00167-009-0989-4 20012014

[B39] Cole J Brand JC Jr Caborn DN Johnson DL Radiographic analysis of femoral tunnel position in anterior cruciate ligament reconstruction Am J Knee Surg 2000 13 218 222 11269541

[B40] Kondo E Yasuda K Ichiyama H Azuma C Tohyama H Radiologic evaluation of femoral and tibial tunnels created with the transtibial tunnel technique for anatomic double-bundle anterior cruciate ligament reconstruction Arthroscopy 2007 23 869 876 10.1016/j.arthro.2007.02.016 17681209

[B41] Belisle AL Bicos J Geaney L Strain pattern comparison of double- and single-bundle anterior cruciate ligament reconstruction techniques with the native anterior cruciate ligament Arthroscopy 2007 23 1210 1217 10.1016/j.arthro.2007.06.021 17986409

[B42] Mae T Shino K Miyama T Single- versus two-femoral socket anterior cruciate ligament reconstruction technique: Biomechanical analysis using a robotic simulator Arthroscopy 2001 17 708 716 10.1053/jars.2001.25250 11536089

[B43] Morimoto Y Ferretti M Ekdahl M Smolinski P Fu FH Tibiofemoral joint contact area and pressure after single- and double-bundle anterior cruciate ligament reconstruction Arthroscopy 2009 25 62 69 10.1016/j.arthro.2008.08.014 19111220

[B44] Petersen W Zantop T Anatomy of the anterior cruciate ligament with regard to its two bundles Clin Orthop Relat Res 2007 454 35 47 17075382 10.1097/BLO.0b013e31802b4a59

[B45] Tsai AG Wijdicks CA Walsh MP Laprade RF Comparative kinematic evaluation of all-inside single-bundle and double-bundle anterior cruciate ligament reconstruction: A biomechanical study Am J Sports Med 2010 38 263 272 10.1177/0363546509348053 19966094

